# Fast, Bright, and Reversible Fluorescent Labeling of Rhodamine-Binding Proteins

**DOI:** 10.1021/jacs.5c18083

**Published:** 2025-12-30

**Authors:** Julian Kompa, Lars J. Dornfeld, Nicola Porzberg, Soohyen Jang, Silja Zedlitz, Simon H. Lilje, Claudia Catapano, David Jocher, Lukas Merk, Carsten Hoege, Runyu Mao, Jonas Wilhelm, Marina S. Dietz, Miroslaw Tarnawski, Julien Hiblot, Anthony A. Hyman, Mike Heilemann, Kai Johnsson

**Affiliations:** † Department of Chemical Biology, 28296 Max Planck Institute for Medical Research, Heidelberg 69120, Germany; ‡ Institute of Physical and Theoretical Chemistry, 9173 Johann Wolfgang Goethe University, Frankfurt Am Main 60325, Germany; § IMPRS on Cellular Biophysics, Frankfurt Am Main 60438, Germany; ∥ 28271 Max Planck Institute of Molecular Cell Biology and Genetics, Dresden 01307, Germany; ⊥ Max Planck School Matter to Life, Heidelberg 69120 Germany; # Protein Expression and Characterization Facility, Max Planck Institute for Medical Research, Heidelberg 69120, Germany; ∇ Institute of Chemical Sciences and Engineering (ISIC), École Polytechnique Fédérale de Lausanne (EPFL), Lausanne CH-1015, Switzerland

## Abstract

Rhodamine dyes conjugated to targeting ligands can yield exceptionally bright fluorescent probes for live-cell imaging. However, the limited permeability of such rhodamine derivatives restricts their broader applications, particularly *in vivo*. Here, we present Rho-tag and SiR-tag, engineered protein tags derived from bacterial multidrug-resistant proteins that bind unsubstituted (silicon) rhodamines with nanomolar affinity. Unsubstituted (silicon) rhodamines readily cross membranes and enable rapid, reversible, and fluorogenic labeling of the tags in mammalian cells within seconds. The labeling of Rho-tag and SiR-tag is compatible with various super-resolution imaging methods and allows their use alongside self-labeling tags, such as HaloTag7 and SNAP-tag. The high affinity and specificity of both tags, combined with the permeability and outstanding spectroscopic properties of rhodamines, make them particularly attractive for *in vivo* bioimaging, as demonstrated by efficient fluorescent labeling in*C. elegans*embryos and zebrafish larvae.

## Graphical abstract

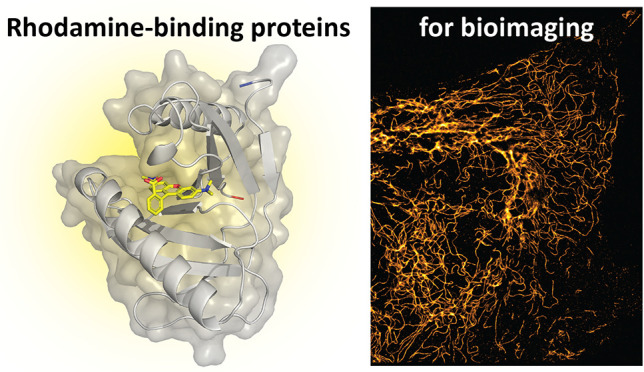

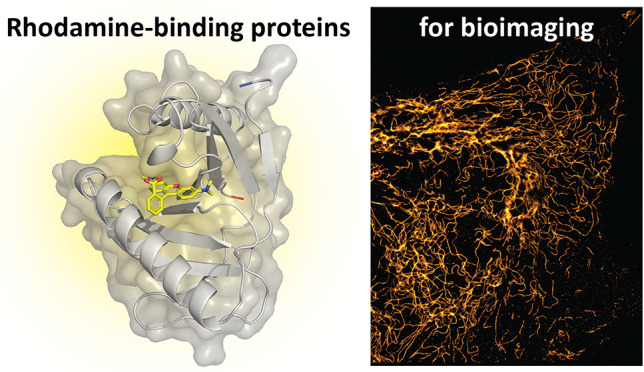

## Introduction

The development of fluorescent probes and labeling strategies has been fundamental to advances in bioimaging.^1^ Fluorescent proteins (FPs) remain the most popular fluorescent probes, as they can be genetically targeted to the protein or cell of interest.^2^ Synthetic fluorophores, particularly those in the red and far-red spectral regions, are brighter and more photostable than FPs but require advanced labeling strategies.^3^ Among the chemical dyes, rhodamine probes stand out due to their excellent brightness, cell permeability, and broad spectral range.^4^ Another key feature is that they exist in an environmentally sensitive equilibrium between a fluorescent zwitterion (open) and a non-fluorescent (closed), yet cell-permeable spirolactone form.^5^ This has been extensively explored for the generation of fluorogenic rhodamine derivatives.^6,7^ Rhodamine probes are typically targeted using self-labeling protein (SLP) tags like SNAP-tag^8^ or HaloTag.^9^ While these tags enable relatively fast and efficient covalent labeling of fusion proteins, they require the derivatization of rhodamines with a substrate for the SLPs, such as the HaloTag Ligand (HTL). The resulting rhodamine derivatives are relatively large molecules (molecular weight >500 Da) with limited permeability. As a consequence, labeling *in vivo* even at high doses is relatively inefficient and slow, even for the most permeable rhodamines.^10,11^ An alternative to the labeling of SLPs with rhodamines is the use of proteins that directly bind underivatized fluorophores with high binding affinity like fluorogen-activating proteins (FAPs); however, these systems have been predominantly applied to extracellular targets.^12^ In contrast, the fluorescence-activating and absorption-shifting tag (FAST) enables live-cell imaging by targeting FP-like fluorogens of different colors using a single protein tag.^13^ However, the spectroscopic properties of the underlying fluorophores are inferior to those of rhodamines.

Here, we present two rhodamine-binding protein tags (Rho-tag and SiR-tag) for live-cell and *in vivo* protein labeling with tetramethylrhodamine (TMR) and silicon rhodamine (SiR). Rho-tag and SiR-tag bind their substrates with nanomolar binding affinity, high specificity, and speed. Binding of TMR and SiR by their respective tags improves the brightness of the fluorophores, and Rho-tag and SiR-tag can be used in super-resolution microscopy, including points accumulation for imaging in nanoscale topography (PAINT),^14^ single-particle tracking (SPT),^15^ stimulated emission depletion (STED) microscopy,^16^ and MINFLUX.^17^ Due to their fast labeling and good permeability, TMR and SiR efficiently label the two tags in bacteria, cultured mammalian cells, nematode embryos, and zebrafish larvae, opening up new applications for rhodamines in bioimaging.

## Results

### Candidate Selection and Rho-tag Engineering

*In silico* pharmacokinetic analysis of TMR-HTL (in its open form) and SiR-HTL (in its closed form, Figure 1A) using the SwissADME^18^ web tool suggests that these molecules possess relatively poor brain penetration (Figure 1B, Table S1) which is in line with previously reported observations.^10,11^ However, the unsubstituted fluorophores without any HTL (Figure 1A) were predicted to readily cross the blood–brain barrier (BBB). This suggests that a protein with a high affinity for unsubstituted rhodamines, like TMR or SiR, could be advantageous for labeling applications *in vivo* in general.

**Figure 1:**
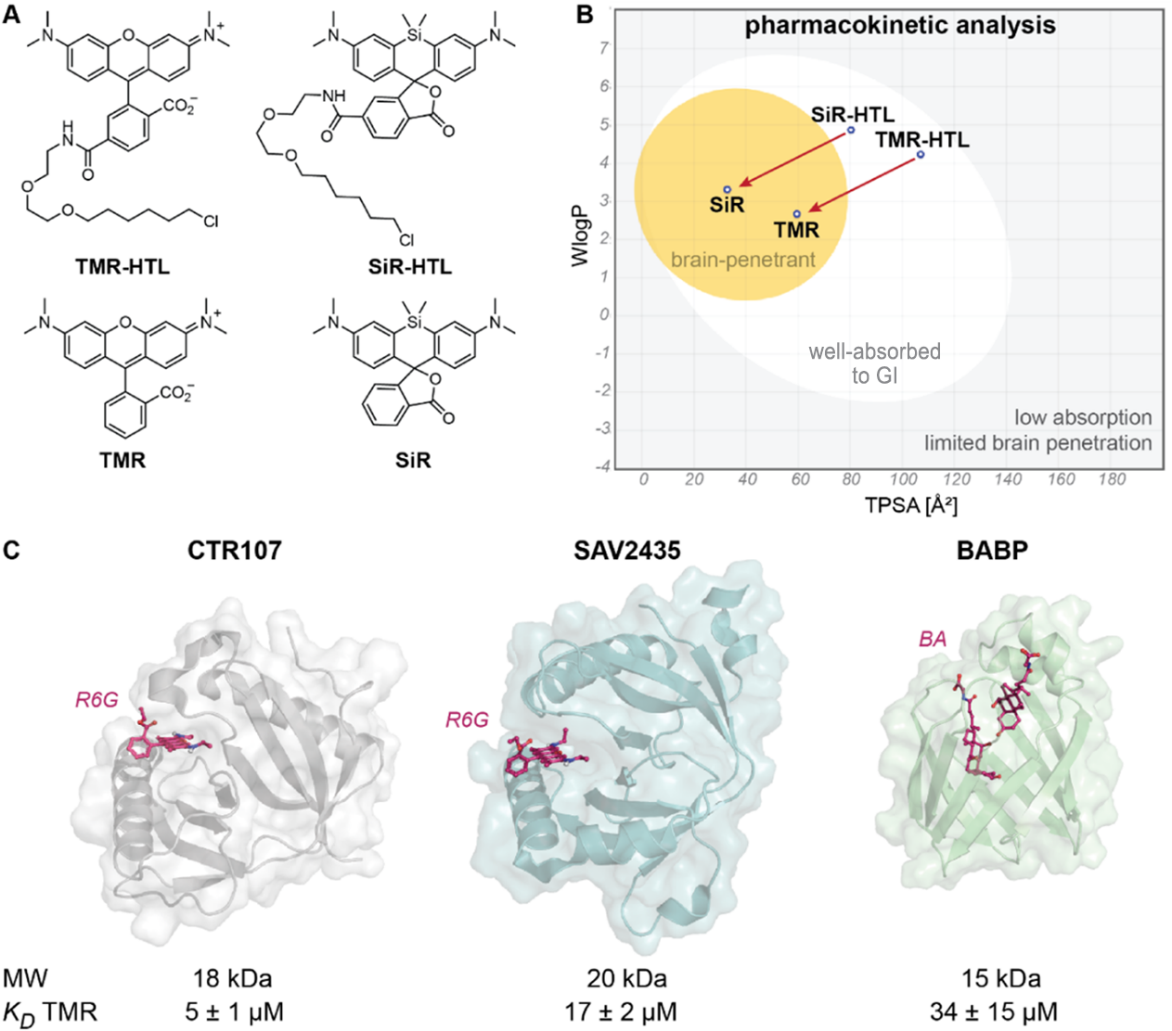
Dye and candidate selection for the development of Rho-tags. A) Chemical structure of TMR and SiR and their corresponding HaloTag Ligand (HTL) derivatives. SiR is shown in its closed form (spirolactone), whereas TMR is shown in its open zwitterionic form. B) BOILED-Egg^54^ representation generated with SwissADME^18^ for molecules shown in (A). Correlation between the topological polar surface area (TPSA) and predicted water partitioning coefficient (WlogP). Gray area—predicted low passive absorption to the gastrointestinal (GI) system and limited brain access (BBB). Egg white—high probability for GI absorption. Egg yolk—high probability for BBB permeation. Blue dot—substrate is predicted to be actively exported from the central nervous system. C) Ligand-bound structures, size (MW), and binding affinity to TMR (*K*_D_) of three potential Rho-tag candidates. Two multidrug binding proteins CTR107 (PDB-ID5KAX, X-ray, 2.0 Å resolution) and SAV2435 (PDB-ID5KAW, X-ray, 1.8 Å resolution) in complex with Rhodamine 6G (R6G). Bile-acid binding protein (BABP, PDB-ID2LFO, NMR) in complex with bile acids (glycochenodeoxycholic and glycocholic acids, BA). Tertiary structures are represented as colored cartoons and a surface. The ligands are represented as purple sticks. The binding affinity *K*_D_ was derived from fluorescence polarization experiments at constant dye concentration (20 nM) and serial protein dilutions (0 – 200 μM). Average data and standard deviation from at least three independent measurements (*N* ≥ 3). CTR107 was selected as the lead candidate for the development of Rho-tags due to its moderate binding affinity, small size, and single-domain state.

We identified three proteins known to bind rhodamines from literature resources and the Protein Data Bank (PDB; Figure 1C).^19^ The multidrug-resistant (MDR) proteins CTR107 and SAV2435 display a globular fold of 18 and 20 kDa, respectively, and have been cocrystallized with rhodamine 6G (R6G).^20^ The bile acid-binding protein (BABP) has been exploited to encapsulate rhodamine dyes on functionalized surfaces.^21^ These three proteins were recombinantly expressed and evaluated for their TMR binding. SAV2435 and BABP showed weak binding to TMR (*K*_D_ = 17 ± 2 and 34 ± 15 μM, respectively). In addition, SAV2435 quenched the TMR fluorescence, presumably through a tryptophan residue in the binding site (Figure S1). CTR107 displayed a *K*_D_ of 5.2 ± 0.7 μM for TMR, which made it the most promising scaffold for the development of a rhodamine binder. We tested the binding affinity of CTR107 for a panel of standard dyes and found that it binds to different rhodamines, such as rhodamine B and R101, and pyronines (Figure S2). However, compared to TMR and SiR, these dyes displayed a strong cellular background when incubated with U2OS cells, restricting their use for live-cell fluorescence imaging (Figure S3). We therefore focused on the generation of high-affinity TMR and SiR binders.

Next, we performed a structure-guided site-directed mutagenesis screening on CTR107 (Figure S4) and identified the mutations E36V and N138A, which improved the TMR binding by 48-fold (*K*_D_ 108 ± 29 nM). This first-generation Rho-tag0.1 was expressed in live U2OS cells as a histone 2B (H2B) fusion and could be specifically labeled with TMR, which was not possible with the parental CTR107 (Figure S5). As we observed minor background staining from unbound fluorophore, we attempted to further increase the binding affinity of Rho-tag0.1 by directed evolution (Supplementary Note 1, Figure S6A) to enable imaging at lower dye concentrations. Two types of Rho-tag0.1 libraries were displayed on the surface of yeast and subjected to iterative rounds of fluorescence-activated cell sorting (FACS). First, we generated site-saturation mutagenesis libraries (SSMLs) targeting the 16-amino-acid α-helix *Hα1* (^31^LGSLFVAGYHDILQLL^46^; Figure S6B) near the fluorophore-binding site. From these libraries, we identified the double mutation S33N/H40T, which further improved the binding affinity toward TMR by 6-fold (*K*_D_ 19 ± 9 nM). Second, we screened a synthetic deep mutational scanning (DMS) library covering the full protein, which displayed high enrichment of the M64F mutation (Supplementary Note 1). To offset a decrease in thermostability observed throughout the engineering process, we employed the Protein Repair One-Stop Shop (PROSS)^22^ to generate protein variants with increased thermostability while retaining similar binding affinities (Supplementary Note 2, Table S2). Combining the previously discovered E36V/N138A with the S33N/H40T, M64F mutations, and 14 additional mutations suggested by PROSS yielded the final Rho-tag with a melting temperature of 61 °C.

### Rho-tag is a Bright and Specific Binder to TMR for Cellular Labeling

Rho-tag possesses strong binding affinity for TMR (*K*_D_ = 2.7 ± 2.1 nM, Figure 2A), an ∼2000-fold improvement relative to parental CTR107 (Figure S6C). The *N*-terminal cysteine residues (positions 5 and 9) can be mutated to serine without impairing the binding (Supplementary Note 3), which might be useful for applications in an oxidative environment such as the cell surface. We further investigated the binding kinetics of TMR *in vitro*. Stopped-flow fluorescence anisotropy experiments revealed on-rates close to the diffusion limit (*k*_on_ 1.4 ± 0.5 × 10^8^ M^–1^s^–1^, Figure 2A). The off-rate *k*_off_ of TMR was calculated from *K*_D_ and *k*_on_ as 0.4 ± 0.3 s^–1^. The quantum yield of TMR increased upon binding to Rho-tag from 0.44 ± 0.01 to 0.73 ± 0.02, resulting in a 2-fold increase in brightness (Figure 2B), which we attribute mainly to the efficient suppression of nonradiative decay pathways, such as twisted intramolecular charge transfer (TICT).^23^ This makes the Rho-tag about 36% brighter than TMR-labeled HaloTag7 *in vitro* (Figure 2B). Rho-tag can also be labeled with JF_549_,^23^ which shows similar binding affinity to TMR (*K*_D_ 4.0 ± 1.2 nM) with a further enhanced quantum yield (0.87 ± 0.02, Table S3).

**Figure 2:**
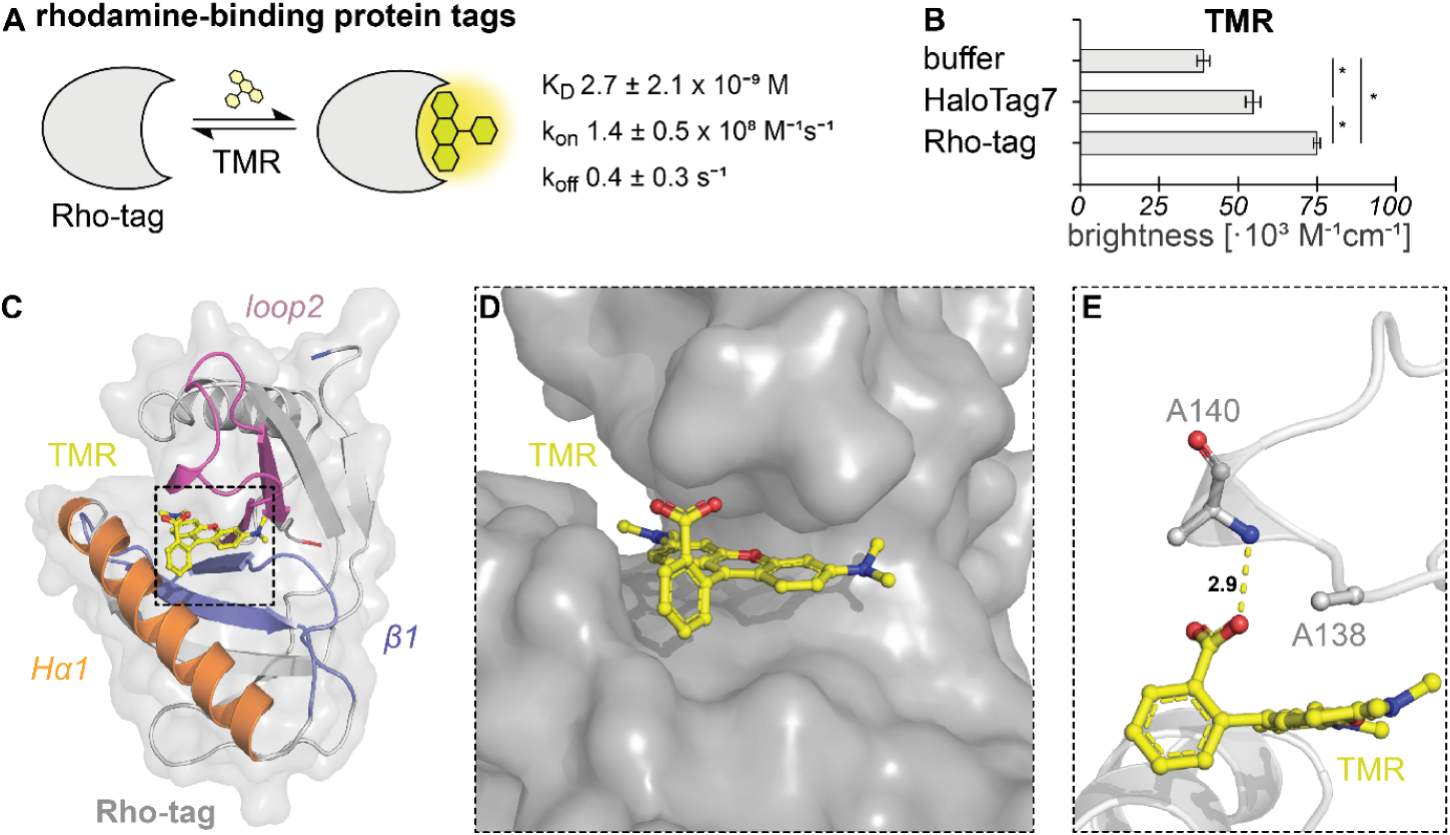
Fast, bright, and reversible binding of TMR to Rho-tag. A) Schematic representation of Rho-tag binding to TMR and binding parameters. *K*_D_ was determined via fluorescence polarization (TMR, 0.5 nM) from *N* = 12 independent measurements. *k*_on_ was measured via stopped-flow anisotropy. *k*_off_ was calculated from the values of *K*_D_ and *k*_on_. B) Molecular brightness (ε × Φ) of TMR compared between unbound, Rho-tag-bound, or HaloTag7-bound states. The absolute quantum yield (Φ) and extinction coefficient (ε) were measured in the absence or presence of 20 μM Rho-tag and multiplied. Average data from *N* = 3 replicates ± SD. Significance was calculated using two-sided *t*-tests. n.s.: *p* ≥ 0.05, *: *p* < 0.05. C) Crystal structure of Rho-tag in complex with TMR (PDB-ID9RTM, 2.1 Å resolution). Tertiary structures are represented as colored cartoons and surface. Secondary element sites of engineering are colored according to the legend. The TMR ligand is presented as yellow sticks. D) Surface representation of Rho-tag bound to the TMR ligand (sticks) shows insertion of the xanthene ring into the binding pocket. E) Hydrogen bonding between the TMR *ortho*-carboxylate and the α-amino group of A140. The space needed to bind TMR tightly was created by the N138A mutation. Distance is measured in Å.

To understand the molecular basis of ligand binding, we determined the crystal structure of the Rho-tag in complex with TMR at 2.1 Å resolution (Figure 2C, PDB-ID9RTM), which reveals a deep insertion of the xanthene moiety into the binding pocket (Figure 2D). We found a putative hydrogen bond interaction between the α-amino group of A140 and the *ortho*-carboxy moiety of TMR. We assume that this interaction is important to stabilize the dye in its open, fluorescent form (Figure 2E). We further performed classical molecular dynamics (MD) simulations using the Rho-tag/TMR complex crystal structure. The ligand RMSD (root-mean-square deviation) remains relatively constant (∼0.3 Å) throughout the simulation. All rotatable bonds are conformationally restrained (Figure S7), providing a rationale for the high quantum yield.

Rho-tag could be labeled with TMR in live *E. coli* (Figure S8) and mammalian U2OS cells (Figure 3A). The high affinity for TMR allowed the use of dye concentrations as low as 50 nM in these experiments, which resulted in a very low background signal. When fused to H2B, the signal-to-background ratio (S/B) and signal brightness, normalized to a cotranslational mEGFP expression marker via a T2A sequence,^24^ was determined to be similar to that of HaloTag7 (Figure 3A, B). Both live-cell and fixed-cell labeling yielded bright fluorescent signals (Figure 3B, Figure S9A). Washing TMR-labeled cells gradually removed the bound rhodamine (Figure S9B). These cells can be labeled and washed repetitively, demonstrating the reversibility of the labeling. Full labeling of nuclear-localized Rho-tag-mEGFP-NLS_3_ in live cells was reached within the first recorded frame (∼30 s, Figure 3C, D), whereas complete labeling of HaloTag7 under the same conditions required approximately 10 min (half-labeling time of 5.6 min). This makes the labeling of Rho-tag at least 10-times faster than that of HaloTag7 in the same cell type. We attribute the faster in-cell labeling of Rho-tag to its superior labeling kinetics^25^ and the improved permeability of TMR compared to TMR-HTL. Bleaching experiments showed similar photostability compared to covalently labeled HaloTag7 (Figure S10). Rho-tag could be localized to various cellular structures like the endoplasmic reticulum or intermediate filaments (Figure 3E). Finally, we investigated if Rho-tag can be multiplexed with established SLPs: linking an (exchangeable) HaloTag or SNAP-tag ligand via carbon 6 of the benzyl ring of TMR reduced the binding affinity to Rho-tag by up to 7000-fold (Table S4). Finally, we demonstrate the imaging of two tags with rhodamines within one cell (Figure 3F).

**Figure 3:**
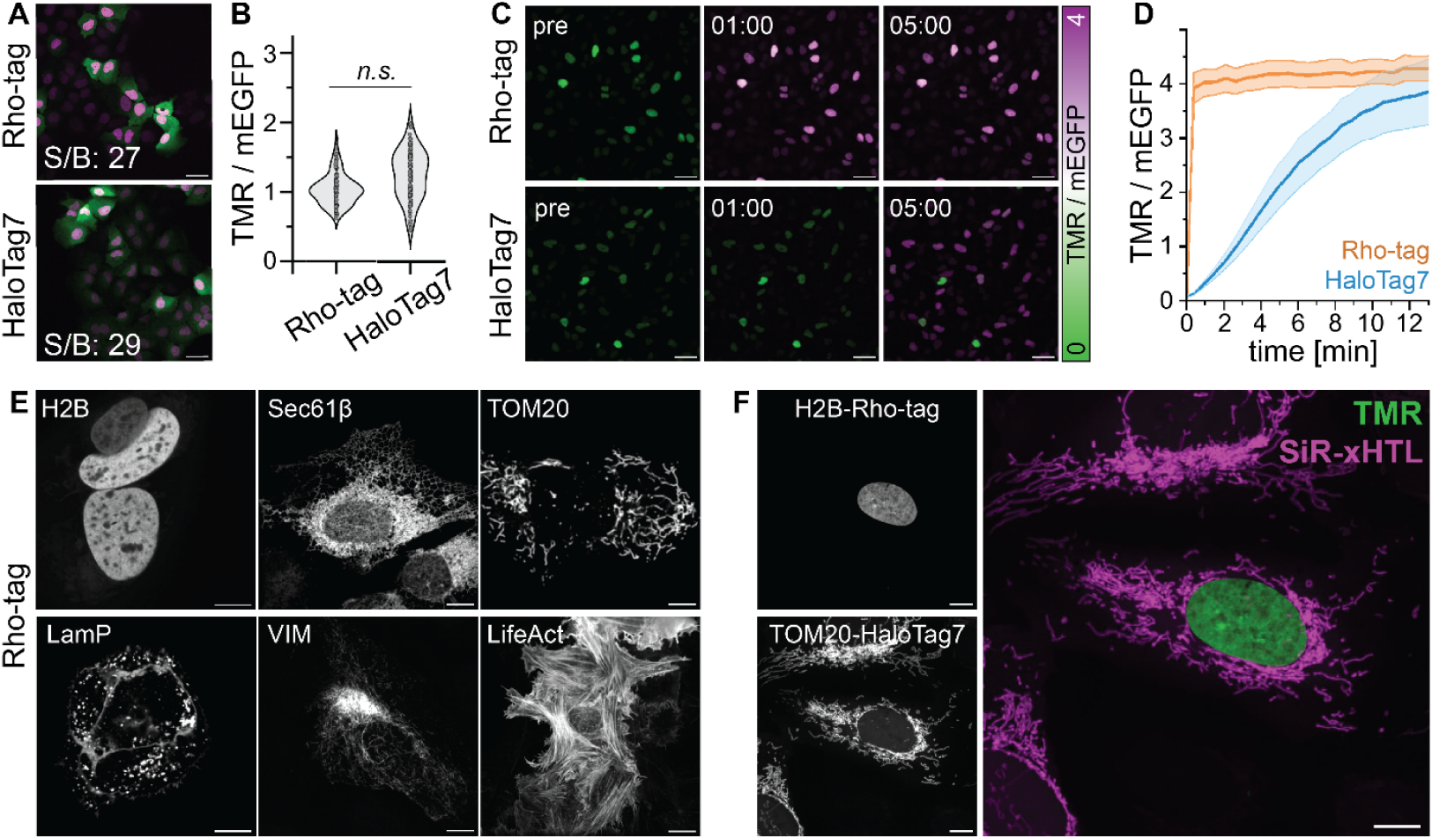
Live-cell labeling characterization of the Rho-tag with TMR. A) Fluorescence micrographs (max. projections) of live U2OS cells expressing H2B-Rho-tag or -HaloTag7 labeled with TMR or TMR-HTL, respectively (50 nM). Cotranslational mEGFP expression (T2A) was used to identify transgene-expressing cells. Signal-overbackground (S/B) ratios calculated as the average nuclear fluorescence intensity from cells expressing Rho-tag/HaloTag7 over non-expressing cells. Scale bars: 50 μm. B) Cellular intensity comparison between Rho-tag and HaloTag7 labeling with TMR in live cells from similar images as in A). More than 100 nuclei were quantified from 3 images, global background subtracted, and TMR signal divided by the individual mEGFP signal. Significance was calculated using two-sided *t*-tests including the Welch correction. n.s.: *p* ≥ 0.05, *: *p* < 0.05. C) Live-cell labeling kinetics of Rho-tag and HaloTag7. U2OS cells expressing Rho-tag or HaloTag7-P_30_-mEGFP-NLS_3_ were labeled with TMR or TMR-HTL (50 nM), respectively. Scale bars: 50 μm. D) Signal and standard error from experiments as shown in C. Quantified from >60 nuclei normalized to the mEGFP signal. Live-cell labeling half-time: Rho-tag/TMR <30 s, HaloTag7/TMR-HTL 5.6 min. E) Overexpression (CMV promoter) of Rho-tag fusions to the following marker proteins: histone—H2B, endoplasmic reticulum—Sec61β, outer mitochondrial membrane—TOM20, lysosomes—LamP1, intermediate filaments—Vimentin (VIM), actin—LifeAct. Max. projections. Scale bars: 10 μm. F) Live-cell confocal multiplexing of H2B-Rho-tag/TMR (50 nM, nucleus, stable cell-line) and TOM20-HaloTag7/SiR-xHTL (500 nM, mitochondria, rAAV). Maximum projection. Scale bar: 10 μm.

### Circular-Permuted and Bioluminescent Rho-tag Variants

The original *N*- and *C*-termini of the Rho-tag are relatively distant from the fluorophore-binding site (26 and 30 Å, respectively). Having the termini close to the site of the fluorophore can be advantageous for the generation of biosensors.^26^ To achieve this, we connected the original termini with a designed linker, using RFdiffusion for backbone generation and ProteinMPNN for sequence design,^27–29^ and introduced new termini at positions 64/67 (Supplementary Note 4). This yielded cpRho-tag, which offered high thermostability (60 °C) and retained its high binding affinity for TMR (*K*_D_ 6.8 ± 5.8 nM). In live-cell labeling experiments, the cpRho-tag performed as well as the Rho-tag (Figure S11). cpRho-tag should be useful for insertion of the tag into protein structures.

Bioluminescence reporters based on bioluminescence resonance energy transfer (BRET, Figure 4A) have gained popularity in bioimaging and biosensing due to their high sensitivity.^30^ We either fused NanoLuc (NLuc) to the *N*- or *C*-terminus of Rho-tag or inserted a circular-permuted version of NLuc^31^ at position 64/67 of Rho-tag to create RhoLuc (Figure 4B). Upon the addition of TMR and the luciferase substrate, we detected a bioluminescent signal (Figure 4C) with an emission maximum at 593 nm. The chimeric RhoLuc displayed four times higher BRET efficiency (*I*_593_/*I*_459_ nm) than the terminal fusions.

**Figure 4:**
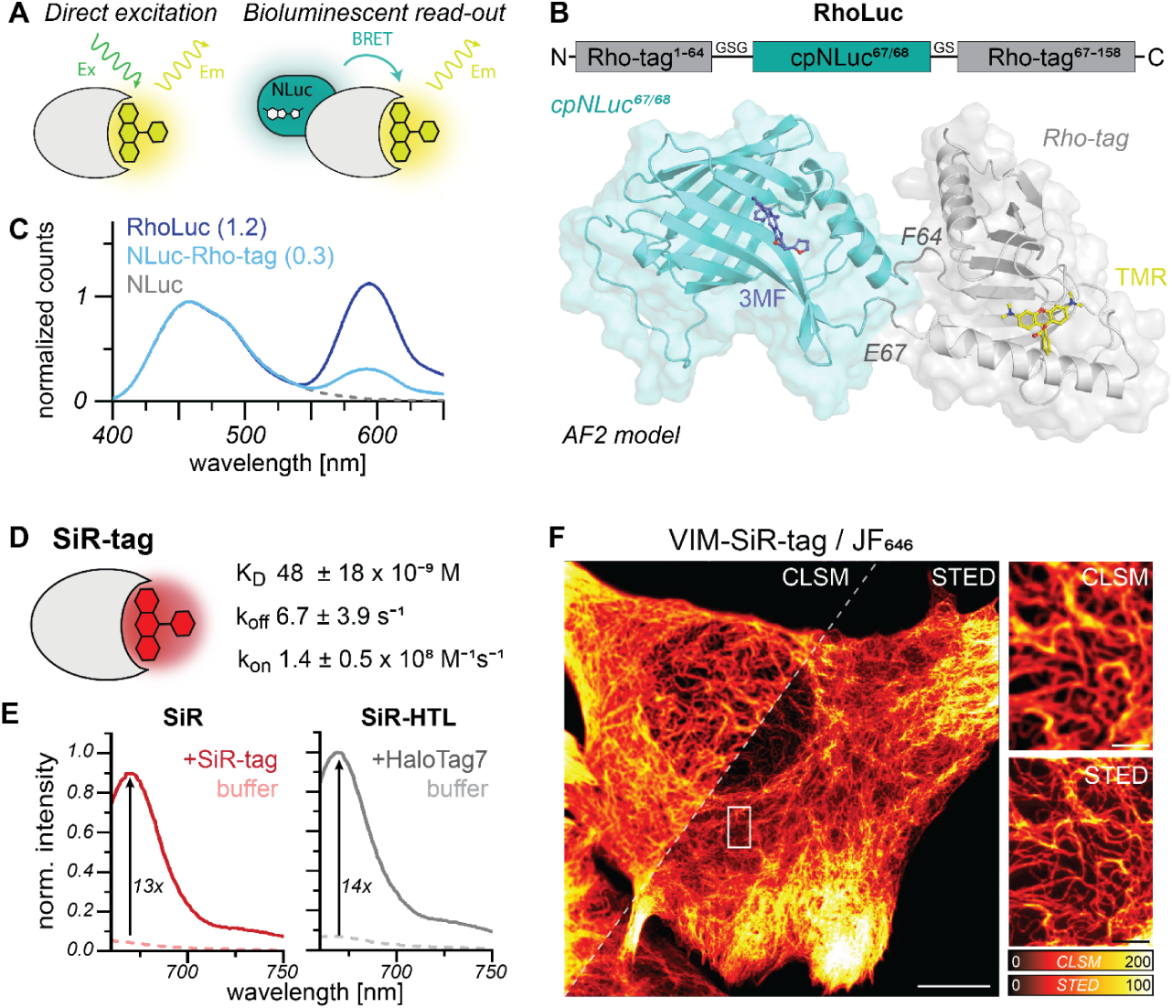
Bioluminescent Rho-tag and far-red SiR-tag. A) Schematic representation of direct excitation (left) or bioluminescence (BRET, right) of Rho-tag/TMR. B) AlphaFold2 (AF2) model of RhoLuc (*N*-Rho-tag^1‑64^-GSG-cpNLuc^67/68^-GS-Rho-tag^67‑158^-*C*). cpNLuc^67/68^ was inserted into the 64/67 site of Rho-tag. Tertiary structures are represented as colored cartoons and surface. The TMR ligand presented as yellow sticks. 3-Methoxyfurimazine (3MF) was modeled into the structure by aligning a crystal structure of NLuc (PDB-ID7SNT) to the AF model and is represented as blue sticks. pLDDT: 93.88. C) Bioluminescence profile of RhoLuc (dark blue, insertion), NLuc-Rho-tag (light blue, terminal fusion), or NLuc (gray). Proteins (2 nM) were labeled with excess TMR (50 nM). The fluorescence emission spectra were recorded upon the addition of Nano-Glo substrate (Promega 1:1000). BRET ratio (in brackets) is given as *I*_593_/*I*_459_. Average data from three technical replicates. D) Schematic representation of SiR-tag binding to SiR and binding parameters. *K*_D_ was determined via fluorescence intensity (SiR, 20 nM) assay from *N* = 4 independent measurements, respectively. *k*_on_ measured via stopped-flow anisotropy. *k*_off_ was calculated from *K*_D_ and *k*_on_. E) Fluorescence emission increase of SiR upon binding to SiR-tag and covalent binding to HaloTag7. The fluorescence emission spectrum of 50 nM dye was recorded in the presence and absence of 20 μM protein. *F*_I_/*F*_I0_ indicated by a black arrow. Average data were obtained from technical triplicates. F) Live-cell CLSM and STED microscopy of U2OS cells expressing VIM-SiR-tag and labeled with JF_646_ (500 nM). Scale bars: 10 μm (overview) and 2 μm (magnification). Pixel intensities scaled according to the reference bar.

### SiR-tag: A Fluorogenic Binder for Far-red-Emitting Dyes

SiR is a commonly used fluorophore for bioimaging due to its far-red emission wavelength and excellent fluorogenicity.^32^ However, the Rho-tag displayed only weak binding affinity for unsubstituted SiR (*K*_D_ 6.5 ± 2.1 μM). We used Rho-tag as a starting point to develop a SiR-binding protein tag (SiR-tag), taking advantage of the crystal structure of the Rho-tag (Figure 2C) and applying a two-step directed evolution strategy. First, we randomized positions 133 to 149 close to the xanthene (*loop2*, Figure 2C) by creating libraries that encompassed four randomized positions (Supplementary Note 5). We submitted the libraries to yeast display during five rounds of FACS screening, which yielded eight putative SiR-tag candidates. Among them, the variant with the mutations A138S/P139H/A140Y showed a 72-fold improved binding affinity for the SiR derivative JF_646_ ^23^ (*K*_D_ 92 ± 35 nM). We increased the thermostability of the protein using PROSS and further randomized the positions 51 to 79 (*β1,* Figure 2C), following a similar strategy as described above. This yielded the SiR-tag (Figure 4D) which displayed high binding affinity to JF_646_ (*K*_D_ 20 ± 14 nM) and SiR (*K*_D_ 48 ± 18 nM).

SiR-tag displays high stability (*T*_M_ 72 °C) and fast exchange kinetics, similar to the Rho-tag (Figure 4D). In contrast to TMR, SiR exists predominantly in its spirolactone form.^32^ Binding to SiR-tag reveals a strong fluorescence increase (13-fold), which is comparable to the increase in fluorescence intensity observed upon covalent labeling of HaloTag7 with the same fluorophore (Figure 4E) and suggests a shift of the equilibrium of spirocyclization toward the zwitterion form upon SiR-tag binding. SiR-tag could be labeled with JF_646_ in live U2OS cells expressing the H2B-SiR-tag. The staining revealed specific nuclear localization at low dye concentrations (200 nM) with an ∼5-fold improved S/B as compared to Rho-tag/TMR (Figure S12). We attribute the increased S/B to the reduced background signal of the fluorogenic SiR probes. The fluorescence signal of the SiR-tag normalized to the expression level was 1.5-fold less bright compared to that of HaloTag7 after covalent labeling (Figure S12).

SiR-tag is compatible with live-cell STED microscopy of intermediate filaments (Figure 4F) and can be used together with SLPs like SNAP-tag2 ^33^ (Figure S13). SiR-tag has a 325-fold higher binding affinity for JF_646_ over TMR, whereas the Rho-tag has a 2400-fold higher binding affinity for TMR compared to JF_646_, which opens up the opportunity to label the Rho-tag and SiR-tag simultaneously within one cell (Figure S14).

### Rho-tag and SiR-tag for Advanced Microscopy

The reversible yet fast binding and unbinding of the Rho-tag system enable its use in PAINT microscopy (Figure 5A).^14^ First, we determined the binding time of both Rho-tag and SiR-tag in single-molecule imaging experiments conducted in U2OS cells expressing the monomeric membrane receptor CD86 fused to the Rho-tag or SiR-tag. At low dye concentrations (3 nM) and under total internal reflection fluorescence (TIRF) conditions, recurring binding events to single CD86-Rho-tag proteins were detected as single-molecule “blinking” (Video S1), which allowed us to resolve single CD86 clusters (Figure 5B). The localization precisions of 13.1 ± 1.0 nm (Rho-tag) and 9.3 ± 1.6 nm (SiR-tag) were determined using a nearest-neighbor approach.^34^ Average photon counts were 1540 ± 170 (Rho-tag) and 2050 ± 220 photons per frame (SiR-tag), respectively. From super-resolved images, binding times of 951 ± 85 ms (Rho-tag) and 337 ± 11 ms (SiR-tag) were determined, respectively. Thereby we confirmed the fast unbinding with off-rates (*k*_off_) of approximately 1 s^–1^ (Rho-tag) and 3 s^–1^ (SiR-tag, Table S5) required for PAINT microscopy.^35^ To demonstrate the use of Rho-tag as a tool for super-resolution imaging of subcellular structures, we resolved the intermediate filaments with a subdiffraction resolution of 21 nm in chemically fixed U2OS cells expressing Vimentin-Rho-tag (Figure 5C). Similarly, we performed 2D-MINFLUX imaging to resolve single Vimentin molecules along the intermediate filaments in fixed cells with a localization precision of 2.2 nm (Figure 5D).

**Figure 5:**
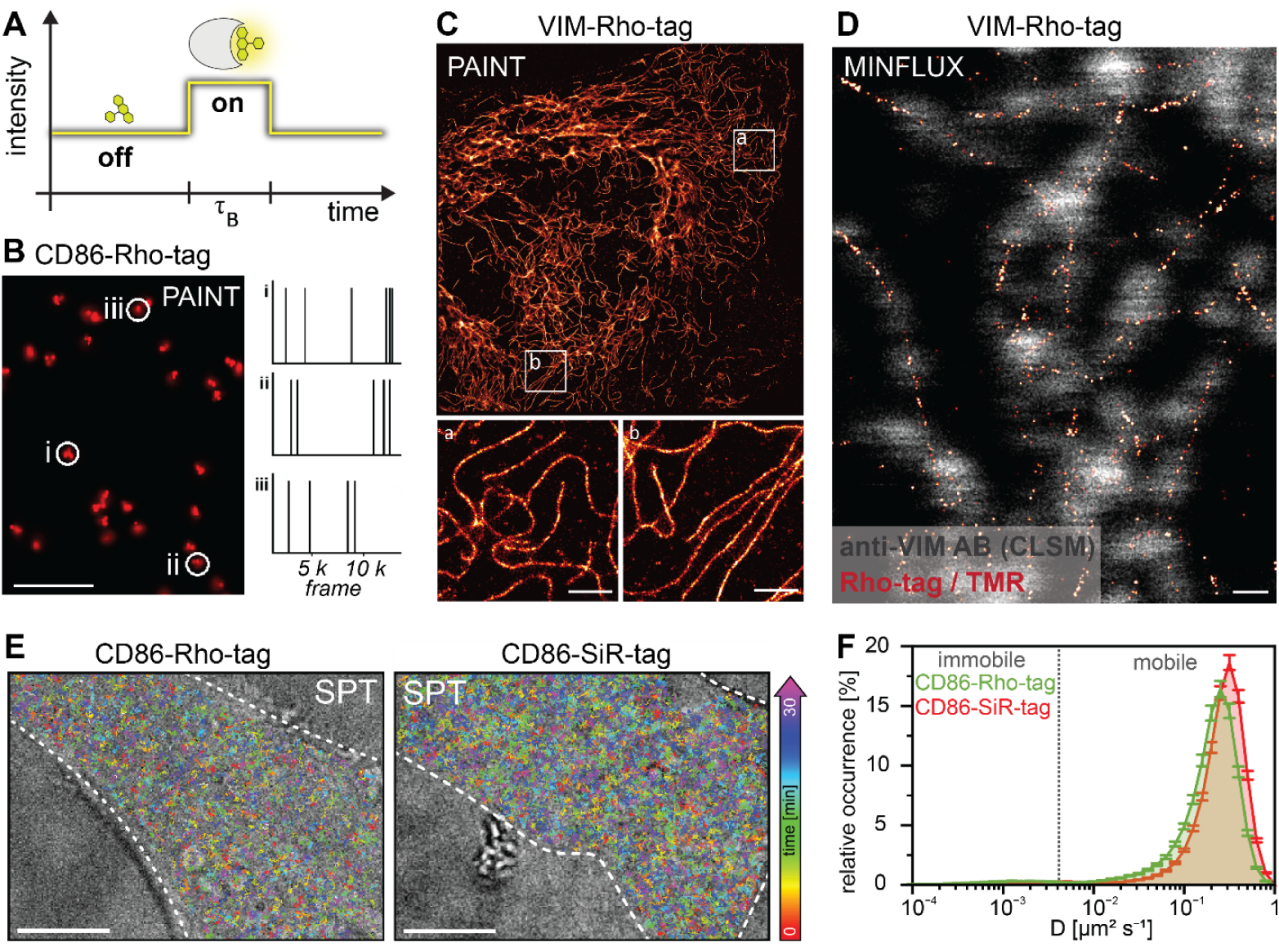
Rho-tag and SiR-tag applications for single-molecule imaging. A) Rho-tag-PAINT imaging concept. Transient binding of TMR to Rho-tag generates a local signal (single-molecule traces) during the binding time τ_B_ and until spontaneous unbinding of the dye. B) Rho-tag-PAINT imaging with TMR (3 nM) on fixed U2OS cells resolved single CD86-Rho-tag clusters with a localization precision of 13.1 nm. Scale bar: 0.5 μm. Single-molecule traces of highlighted clusters yield a binding time of 1.05 ± 0.01 s. C) Super-resolution reconstruction of Vimentin filaments (25,000 frames, 150 ms exposure time) using Rho-tag and TMR (1 nM) in U2OS cells. The resolution was determined using decorrelation analysis^55^ (21 nm). Scale bars: 10 μm (overview) and 2 μm (magnification). D) 2D-MINFLUX imaging (100 photons last iteration, 2 h) with TMR (0.2 nM) in U2OS Vim-Rho-tag cells with a localization precision of 2.2 nm. Costaining of Vimentin with primary anti-Vimentin and secondary AF647-labeled antibodies. Scale bar: 0.2 μm. E) Single-particle tracking (SPT) in living U2OS cells. Single-molecule trajectories recorded of TMR binding to the CD86-Rho-tag (left) or SiR binding to the CD86-SiR-tag (right) expressed in U2OS cells. Trajectories are color-coded by their time of occurrence as indicated by the colored arrow: early appearing trajectories denoted in red, and late appearing trajectories colored in purple. Mean trajectory lifetime per cell (0.65 ± 0.01 s) at a laser power of 0.5 kW/cm.^2^ Scale bar: 10 μm. F) Histogram of the relative abundance of diffusion coefficients revealing immobile (Rho-tag: 4.22 ± 0.17%, SiR-tag: 3.16 ± 0.12%) and mobile (Rho-tag: 95.78 ± 0.17%, SiR-tag: 96.84 ± 0.12%) trajectories across all cells (Rho-tag: *N* = 152, SiR-tag: *N* = 140). Error bars represent SEM. No significant differences in diffusion coefficients or mobility mode between the Rho-tag and SiR-tag were detected (statistical analysis see Supporting Informations).

Live-cell super-resolution imaging with high temporal resolution can be achieved by combining exchangeable probes and high-density single-molecule localization using a neural network^36^ which has been shown to resolve cellular features under physiologically relevant time scales.^37^ We measured single-molecule data of Rho-tag-Sec61β at high density and used a DeepSTORM model from previous work^37^ to reconstruct the movement of the ER by live-cell PAINT (Video S2, Figure S15).^37^ Another powerful single-molecule imaging method in living cells is single-particle tracking (SPT). The integration of exchangeable probes has recently been shown to report diffusion coefficients and dynamic clustering of membrane proteins in long-term imaging experiments.^38^ Using living U2OS cells expressing CD86 fused to Rho-tag or SiR-tag (Figure 5E), a high number of single-molecule trajectories was recorded in comparison to nontransfected U2OS cells (Figure S16A), of which >95% were classified as mobile based on their diffusion coefficient (Figure 5F). The average trajectory lifetime was ∼0.6 s for both protein tags, which is comparable to values reported for other noncovalent labels (Figure S16B).^38^ Over the imaging course of 30 min, the localization density was significantly higher compared to covalent HaloTag7 labeling (Figure S16C), demonstrating the suitability of Rho-tag and SiR-tag for long-term SPT in living cells.

### Rhodamine Tags for *In Vivo* Imaging

TMR and SiR are predicted to readily cross the BBB (Figure 1A) and show fast live-cell labeling kinetics (Figure 3D). We therefore tested their performance *in vivo*. P granules are biomolecular condensates in the germline of *C. elegans* and as such their labeling with a fluorophore within*C. elegans*is challenging, as the dye has to be delivered into the gonads and pass through its basement membrane. We generated*C. elegans*lines with endogenously Rho- or SiR-tagged PGL-3, a P granule^39,40^ scaffolding protein. P granules are crucial for the development of the germline.^41^ The successful homozygous integration was verified via DNA sequencing and anti-PGL-3 immunoblotting (Figure S17). Fluorescent labeling was performed by growing L4 stage worms to young adults in a liquid bacteria/dye culture overnight^42^ and subsequently extracting the stained embryos (Figure 6A).

**Figure 6:**
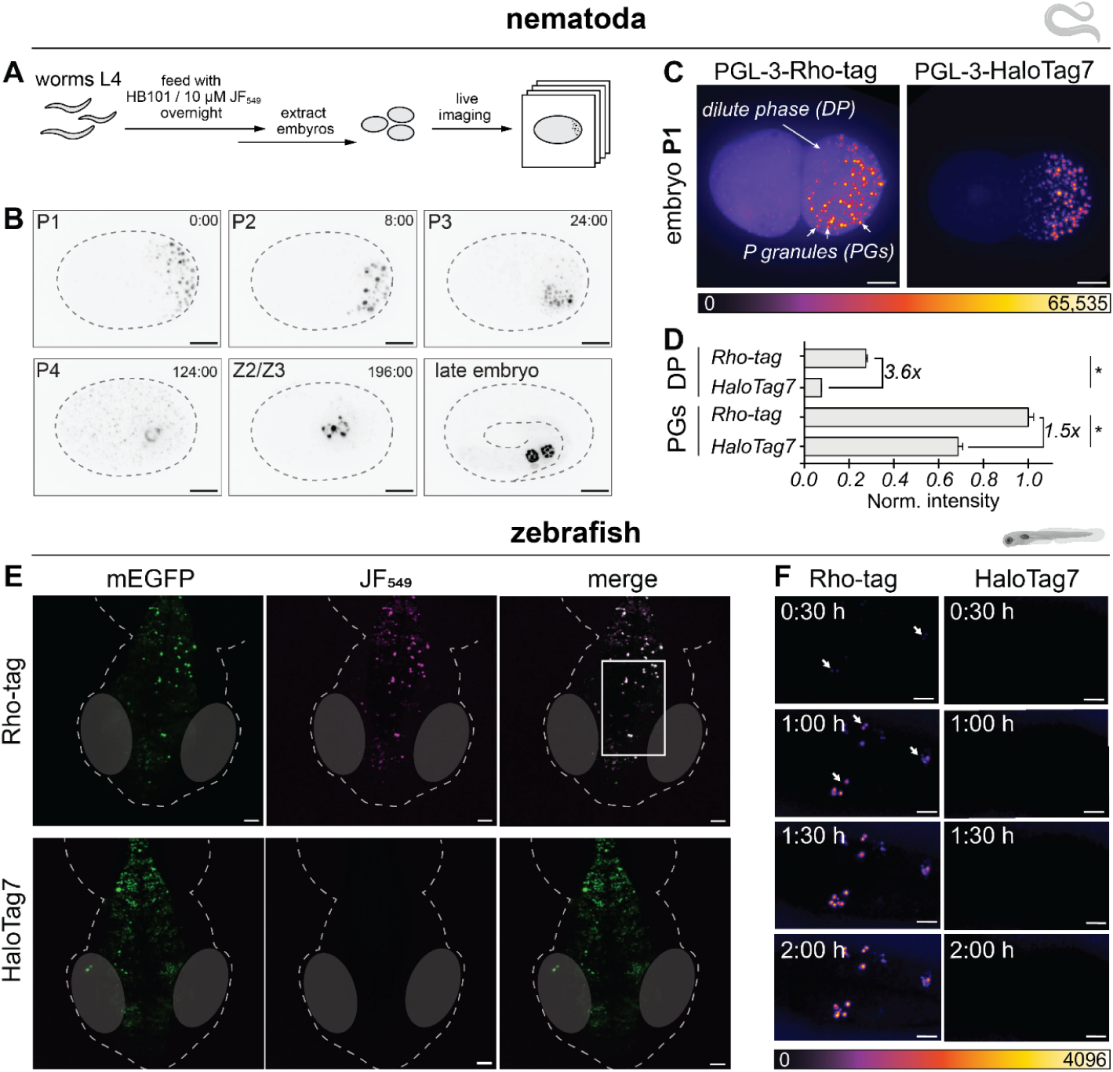
Rho-tag labeling in nematode embryos and zebrafish larvae. A) Schematic workflow for the imaging of Rho-tag in*C. elegans*embryos. Homozygous TH809 PGL-3:Rho-tag strain at the L4 stage was labeled with JF_549_ (10 μM) in the presence of HB101 bacteria with overnight shaking according to published protocols.^42^ Adult worms show background labeling in the gut (data not shown). The embryos were extracted the next day and imaged live. B) Fluorescence micrographs of the germline development of PGL-3:Rho-tag*C. elegans*embryos labeled with JF_549_. Embryo stage^41^ indicated in the top left and the time of recording indicated in the top right (in minutes). Maximum projections of 38 z-stacks (0.8 μm each). The late embryonic stage was acquired individually. Eggshells are marked with dashed lines from brightfield images. Scale bar: 10 μm. C) Fluorescence intensity comparison between PGL-3-Rho-tag and HaloTag7 (P1 stage) labeled with JF_549_ or JF_549_-HTL, respectively. PGL-3 exists in a dilute phase (DP) and segregates into P granules (PGs), as previously reported.^41^ Pixels scaled according to the reference bar (fire LUT). Scale bar: 10 μm. D) Quantification of the fluorescence intensity obtained from PGL-3-Rho-tag or -HaloTag7 in DP or PGs. Signal intensity was quantified from *N* > 60 ROIs from 2 images. Average data and error bars (SEM) presented. Fold improvement of signal intensity between Rho-tag and HaloTag7 labeling shown. Significance was calculated using two-sided *t*-tests. n.s.: *p* ≥ 0.05, *: *p* < 0.05. E) Fluorescence micrographs of zebrafish embryos mosaically expressing neuronal Rho-tag (top) or HaloTag7-P_30_-mEGFP-NLS_3_ (bottom) with the TMR derivative JF_549_ or its respective HaloTag ligand (250 nM, 2 h). Live larvae were embedded in agarose (supplemented with Tricaine) and imaged on a confocal microscope over 200 μm depth (maximum projections). Scale bars: 50 μm. *N* = 6 (Rho-tag) and *N* = 8 (HaloTag7) individual larvae delivered similar results. Zebrafish outlines are marked with dashed lines from brightfield images. F) Live *in vivo* labeling kinetic comparison of Rho-tag and HaloTag7 probes. Inset from (A). Larvae were embedded in agarose, and 250 nM dye was added to the medium. White arrows show the onset of the labeling signal. Pixels scaled according to the reference bar (fire LUT). Scale bars: 10 μm. Figure contains icons from biorender.com.

Rho-tag shows bright and specific labeling of PGL-3, as verified by crossing the previous line with a PGL-3-GFP line^43^ and coimaging of Rho-tag/JF_549_ and GFP (Figure S18A), and virtually no signal in embryos not expressing Rho-tag (Figure S18B). The stable fluorescent labeling of either Rho-tag or SiR-tag allowed us to follow germline development by spinning-disc confocal microscopy and observe the dynamic segregation of PGL-3 from a dilute phase into P granules, as previously reported^41^ (Figure 6B, Video S3, Figure S19). Quantification of the fluorescence signal of PGL-3 in the dilute phase or P granules reveals 3.6- and 1.5-fold brighter signals, respectively, for Rho-tag over HaloTag7 ^44^ labeling using the same fluorophore (Figure 6C,D). We attribute this increased signal brightness to the higher permeability of Rho-tag labels but cannot exclude other factors, such as higher protein stability or labeling efficiency of Rho-tag versus HaloTag7. Overall, these findings imply that labeling experiments with the Rho-tag in *C. elegans* can be performed at markedly lower dye concentrations.

Zebrafish (*Danio rerio*) larvae is a popular model organism in biology,^45^ as their BBB displays structural and functional integrity from 3 to 10 days post-fertilization (dpf).^46^ Therefore, the staining of HaloTag7 in their neurons requires long incubation times (hours) and relatively high dye concentrations (4–30 μM).^10,47,48^ We tested labeling of Rho-tag in neurons of live zebrafish larvae at 3–4 dpf (mosaic expression) and observed a bright and specific signal within 1 h using dye concentrations as low as 250 nM (Figure 6E, F). In contrast, HaloTag7 could not be labeled at these probe concentrations and required overnight staining with 10 μM dye for effective staining (Figure S20). Imaging of zebrafish larvae expressing the Rho-tag and labeled with the TMR derivative JF_549_ was done in the presence of the dye. However, most signals remained stable when the larvae were washed (Figure S21). Similarly, SiR-tag enabled efficient labeling in neurons of live zebrafish larvae. Again, HaloTag7 labeling was less efficient under equivalent conditions using SiR-HTL (Figure S22) or the SiR-variant JF_669_-HTL^49^ (Figure S23). These experiments demonstrate the potential of Rho-tag and SiR-tag for in vivo imaging, as their respective fluorescent labels efficiently cross the BBB.

## Discussion

We introduce two new rhodamine-binding proteins, Rho-tag and SiR-tag, which bind to unsubstituted (silicon-) rhodamines with high binding affinities (nanomolar *K*_D_). Both tags are small and stable single-domain proteins derived from a bacterial MDR protein with a weak intrinsic affinity for rhodamines. Through protein engineering, the affinity of their predecessor toward the fluorophores was increased by about 3 orders of magnitude. The rate of substrate binding to the two tags is close to diffusion controlled. The crystal structure of Rho-tag bound to TMR reveals that the xanthene core is bound deep in the binding pocket of the protein, helping to rationalize the 2-fold increase in brightness of TMR when bound to Rho-tag. The structural information about the dye–protein interaction will aid in future engineering of the tags. Rho-tag can be subjected to circular permutation and allows insertion of other proteins into its structure. For example, we have inserted the luciferase NanoLuc into a loop of Rho-tag such that the fluorophore is close to the active site of the luciferase, creating a chimera that enables a bioluminescent read-out. These experiments suggest that Rho-tag, SiR-tag, and their circularly permutated variants represent an attractive platform for the design of biosensors.

The main applications of Rho-tag and SiR-tag are in live-cell imaging. We demonstrate that the Rho-tag expressed in bacterial and mammalian cells can be labeled with TMR dyes. Labeling of the Rho-tag in mammalian cells occurs within seconds, which is significantly faster than the labeling of self-labeling protein tags such as HaloTag. We assume that the higher cell permeability of unsubstituted TMR, relative to that of the TMR derivate used for HaloTag labeling, contributes to the faster live-cell labeling. Rho-tag can also be labeled in fixed samples, and the labeling of the Rho-tag is reversible, as the fluorophore can be washed out after labeling. For most applications, imaging of Rho-tag and SiR-tag fusion proteins is done in the presence of the dye. The high affinity of the tags for their substrates, which allows their use at nanomolar concentrations, and, in the case of SiR-tag, the fluorogenicity of the used dyes enable live-cell confocal imaging with high contrast. However, the reversibility of Rho-tag and SiR-tag labeling requires, for most applications, the presence of unbound dye. This can lead to a lower S/B compared to covalent labeling, where excess probe can be removed through washing. We note that the reversible binding of TMR to Rho-tag did not result in an increase in photostability when compared to covalent labeling of TMR.^50^ We speculate that the tight binding affinity might prevent efficient exchange of damaged fluorophores or that the close proximity of the dye to the protein might result in photodamage to the binding site. Rho-tag and SiR-tag are compatible with various super-resolution microscopy techniques, including STED, PAINT, and MINFLUX, and show excellent performance in single-particle tracking. Furthermore, as Rho-tag and SiR-tag show very low affinity toward the substrates of the self-labeling proteins HaloTag and SNAP-tag, they can be used in combination with these tags.

The main motivation for the development of Rho-tag and SiR-tag was the assumption that the high permeability of unsubstituted rhodamines should facilitate labeling in complex samples, for example, in the central nervous system of a living animal. Indeed, the Rho-tag expressed in neurons of zebrafish larvae could be efficiently labeled at substrate concentrations at which no labeling of HaloTag7 was observed. The signal of JF_549_-labeled Rho-tag remained stable even after placing the labeled larvae in water in the absence of the dye, which indicates, that the wash out of these dyes is slow enough to enable long-lasting staining in zebrafish larvae (Figure S21). Similarly, the high affinity and resulting stability of the labeling allowed us to record the development of*C. elegans*embryos over the course of hours. Collectively, these experiments highlight the potential of the Rho-tag and SiR-tag for *in vivo* applications.

Rho-tag and SiR-tag are part of a new class of tags that reversibly bind to unsubstituted rhodamines. The other members of this class are *de novo*-designed rhodamine binders,^51,52^ which have similar features to Rho-tag and SiR-tag and have been developed in parallel with this work. These rhodamine-binding tags complement reversible fluorophore tags such as the FAST system,^13^ exchangeable HaloTag Ligands (xHTLs),^50^ or the self-renewable tag (srTAG).^53^ The distinguishing feature of the rhodamine-binding tags, relative to these other tags, is their outstanding spectroscopic properties, such as brightness paired with the excellent permeability of their substrates. Rho-tag and SiR-tag should, therefore, become powerful tools for bioimaging.

## Supplementary Material









## Data Availability

The data supporting the findings of this study are provided within the paper and its Supporting Information. The data are also available from the corresponding authors upon reasonable request. The structures of CTR107^N138A^ and Rho-tag in complex with TMR have been deposited in the Protein Data Bank (PDB-IDs: 9RTL and 9RTM). Plasmids are available on Addgene (ID Nos: 249665–249669, 249671–249672).

## Notes

Detailed methods on protein production and engineering, *in vitro* characterization, cell culture, and microscopy; Video S1: Single-molecule imaging of Vimentin-Rho-tag; Video S2: Super-resolution imaging of Sec61β-Rho-tag; Video S3: Time-resolved confocal imaging of *C. elegans* germline development PGL-3:Rho-tag; Supplementary Notes 1–5 on protein engineering; Supplementary Figures S1–S23, Tables S1–S12, Materials and References; chemical structures; and sequences of used compounds and proteins (PDF)
Single-molecule imaging of Vimentin-Rho-tag labeled with TMR in fixed U2OS cells (AVI)
Super-resolution imaging of Sec61β-Rho-tag labeled with TMR in living U2 OS cells (MP4)
Time-resolved confocal imaging of *C. elegans* germline development (MP4)

## Abbreviations

BABP: bile acid-binding protein
BBB: blood–brain barrier
PDB: protein data bank
Cp: circular-permuted
Dpf: days postfertilization
DMS: deep mutational scanning
FAP: fluorogen-activating proteins
FACS: fluorescence-activated cell sorting
FP: fluorescent proteins
FAST: fluorescence-activating and absorption-shifting tag
H2B: histone 2B
HTL: HaloTag Ligand
JF: Janelia Fluor
MD: molecular dynamics
mEGFP: monomeric enhanced green fluorescent protein
MDR: multidrug resistance
PAINT: points accumulation for imaging in nanoscale topography
PROSS: Protein Repair One-Stop Shop
PyrY: pyronine Y
R6G: rhodamine 6G
Rho-tag: rhodamine-binding protein tag
S/B: signal-overbackground
SiR: silicon rhodamine
SLP: self-labeling protein
SiR-tag: silicon rhodamine-binding protein tag
SSMLs: site saturation mutagenesis libraries
SPT: single-particle tracking
srTAG: self-renewable tag
STED: stimulated emission depletion
TICT: twisted intramolecular charge transfer
TIRF: total internal reflection fluoresence
TMR: tetramethylrhodamine
xHTL: exchangeable HaloTag Ligands
